# Host immune response in returning travellers infected with malaria

**DOI:** 10.1186/1475-2875-11-148

**Published:** 2012-05-03

**Authors:** Gregory MacMullin, Ronald Mackenzie, Rachel Lau, Julie Khang, Haibo Zhang, Nimerta Rajwans, W Conrad Liles, Dylan R Pillai

**Affiliations:** 1 Public Health Ontario, Toronto, ON, Canada; 2 Mount Sinai Hospital, Toronto, ON, Canada; 3 Division of Infectious Diseases, Department of Medicine, University of Toronto, Toronto, ON, Canada; 4 St Michael’s Hospital, Toronto, ON, Canada; 5 Sandra A Rotman Laboratory for Global Health, McLaughlin-Rotman Centre for Global Health, University Health Network, Toronto, ON, Canada; 6 Current address: Departments of Pathology & Laboratory Medicine, Medicine, and Microbiology & Infectious Diseases, The University of Calgary, Diagnostic & Scientific Centre, Room 1W-416, 9-3535 Research Road NW, Calgary, AB, T2L 2K8, Canada

**Keywords:** Malaria, Cytokines, Chemokines, Angiopoietins, Immunology, Severity

## Abstract

**Background:**

Clinical observations suggest that Canadian-born (CB) travellers are prone to more severe malaria, characterized by higher parasite density in the blood, and severe symptoms, such as cerebral malaria and renal failure, than foreign-born travellers (FB) from areas of malaria endemicity. It was hypothesized that host cytokine and chemokine responses differ significantly in CB *versus* FB patients returning with malaria, contributing to the courses of severity. A more detailed understanding of the profiles of cytokines, chemokines, and endothelial activation may be useful in developing biomarkers and novel therapeutic approaches for malaria.

**Materials and methods:**

The patient population for the study (n = 186) was comprised of travellers returning to Toronto, Canada between 2007 and 2011. The patient blood samples’ cytokine, chemokine and angiopoietin concentrations were determined using cytokine multiplex assays, and ELISA assays.

**Results:**

Significantly higher plasma cytokine levels of IL-12 (p40) were observed in CB compared to FB travellers, while epidermal growth factor (EGF) was observed to be higher in FB than CB travellers. Older travellers (55 years old or greater) with *Plasmodium vivax* infections had significantly higher mean cytokine levels for IL-6 and macrophage colony-stimulating factor (M-CSF) than other adults with *P. vivax* (ages 18–55). Patients with *P. vivax* infections had significantly higher mean cytokine levels for monocyte chemotactic protein-1 (MCP-1), and M-CSF than patients with *Plasmodium falciparum*. Angiopoietin 2 (Ang-2) was higher for patients infected with *P. falciparum* than *P. vivax*, especially when comparing just the FB groups. IL-12 (p40) was higher in FB patients with *P. vivax* compared to *P. falciparum*. Il-12 (p40) was also higher in patients infected with *P. vivax* than those infected with *Plasmodium ovale*. For patients travelling to West Africa, IFN-γ and IL-6 was lower than for patients who were in other regions of Africa.

**Conclusion:**

Significantly higher levels of IL-12 (p40) and lower levels of EGF in CB travellers may serve as useful prognostic markers of disease severity and help guide clinical management upon return. IL-6 and M-CSF in older adults and MCP-1, IL-12 (p40) and M-CSF for *P. vivax* infected patients may also prove useful in understanding age-associated and species-specific host immune responses, as could the species-specific differences in Ang-2. Regional differences in host immune response to malaria infection within the same species may speak to unique strains circulating in parts of West Africa.

## Background

Almost half the global population is at risk of contracting malaria, with 300–500 million new clinical cases arising each year that result in between one and three million deaths [1,2]. In countries where malaria is non-endemic, the parasite can still have a significant impact on the population and its healthcare system. When travellers visit malaria endemic regions, especially when visiting friends and relatives (VFR), there is a significant risk of the disease being imported into a non-endemic country [3,4]. Each year, approximately 10,000–30,000 travellers from industrialized nations are expected to contract malaria, and the number is continuing to increase [5]. Toronto, Ontario has a population of 2.48 million people (5.5 million in the Greater Toronto Area), with half of the inhabitants having been born outside Canada [6]. In addition to the large population, over 16 million people visit the city each year [7]. The substantial immigrant population and associated factors create significant potential for malaria cases to occur in the region, despite it being non-endemic.

Severe malaria is defined by the WHO as a situation in which a patient suffering from asexual parasitaemia, with no other obvious causes of symptoms, has one or more clinical or laboratory features that include but are not limited to hyperparasitaemia, impaired consciousness or unrousable coma, renal impairment, and prostration [8,9]. Severe malaria is most often seen in *Plasmodium falciparum*-infected patients, but has been seen, defined under the same criteria, in *Plasmodium vivax-*infected patients [9]. Clinical observations of Canadian-born (CB) travellers suggest they are prone to more severe malaria compared to foreign-born (FB) travellers[10]. It was hypothesized that cytokine, chemokine, and endothelial activation responses would differ significantly between CB and FB travelling patients returning with malaria, as more severe cases of malaria often have characteristically different immune response profiles [11-13]. To address this hypothesis, cytokine, chemokine, and expression of the endothelial activation marker angiopoietin-1/2 (Ang-1/2) was quantified based on CB and FB status, gender, age group, country of travel, and species of *Plasmodium* (*P. vivax, P. falciparum, Plasmodium malariae and Plasmodium ovale)* infection. The resulting data was analysed to identify immune factors associated with disease severity, and other patient sample characteristics.

## Methods

### Ethics approval

The study acquired ethics approval from the Toronto Academic Health Sciences Network Research Ethics Board (REB#10-0094-E).

### Study population and patient inclusion criteria

The malaria specimen database at Public Health Ontario Laboratories (PHOL) comprises patient blood samples received for analysis between 2006 and 2011 coupled to basic patient information. Samples from patients suspected to have malaria were obtained via venipuncture by physicians from hospitals and clinics throughout the Greater Toronto Area and transported to PHOL in EDTA vacutainer (BD) tubes for processing in the clinical parasitology department. Following sample analysis using Giemsa-stained thin and thick smear microscopy, *in vitro* immunochromatographic assays (BinaxNow, Inverness Medical), and quantitative PCR as required, samples positive for malaria were transferred to molecular research for storage and further analysis.

Only samples with complete demographic information, defined as patient age, gender, parasitaemia, country of birth and species of infection, were selected for the study. The sample set was stratified into patients born in Canada, and those who recently immigrated to Canada from malaria endemic regions. The samples from CB patients were considered to be more likely to have severe malaria, as defined by WHO guidelines and the RIFLE criteria for acute renal failure [8,9]. An approximately even number of males and females were selected, with a distribution of ages and parasitaemia. Samples were selected from patients infected with each of the four malaria parasites, generating four distinct sample sets to study. A total of 186 patient samples were analysed in this study. Detailed sample information is depicted in Table 1.

**Table 1:** Detailed sample information, broken down by species of infection, Canadian-born or foreign-born status, sex and gender

	***P. falciparum***	***P. vivax***	***P. ovale***	***P. malariae***
	n = 103	n = 60	n = 14	n = 9
**Canadian-born**	54	12	1	2
**Sex**				
**Male**	34	9	0	0
**Female**	20	3	1	2
**Age**				
**0–18**	6	0	0	0
**19–55**	38	9	1	2
**55+**	10	3	0	0
**Foreign-born (Recent immigrant)**	49	48	13	7
**Sex**				
**Male**	18	26	9	5
**Female**	31	22	4	2
**Age**				
**0–18**	12	8	1	0
**19–55**	30	26	11	6
**55+**	7	15	1	1

### Sample processing and storage

After homogenization through repeated inversions, specimens delivered to Public Health Ontario, which were positive for *P. vivax, P. ovale,* and *P. malariae* were immediately transferred into 110μL whole-blood aliquots. The aliquots were stored in 0.5 mL screw top tubes at −80°C. Specimens positive for *P. falciparum* were first separated into two approximately equal aliquots after homogenizing. One aliquot was further separated into cellular and plasma fractions via centrifugation at 2,000 rpm for 5 min. The plasma fraction and the remaining whole blood aliquot were separated into 110 μL volumes and stored at −80°C in the same manner as the other *Plasmodium spp.*

Patient samples arrived at PHOL along with specimen data sheets filled out by their attending doctor specifying age, gender, travel history, and other personal data. Clinical parasitology test results confirmed species, immunochromatography test results, parasitaemia (parasites/1,000 cells counted), and microscopy as well as PCR observations. The data was de-linked from the patients’ names and other personal identifiers, and the remaining details were added to a computer database, with an original copy of the specimen data sheet being placed under lock and key for reference. Follow-ups with the patients’ physicians by the medical microbiologist occasionally provided more specific patient information.

Samples selected for use in the study required additional processing prior to being used in the assays. All *P. falciparum* samples designated for use in the experiment were from plasma aliquots. The aliquots were removed from −80°C storage, and thawed on ice until they were ready for aspiration. The samples were then separated into two 55μL aliquots and placed back in −80°C storage until required for assays.

Whole-blood samples of *P. vivax, P. ovale, and P.malariae* were removed from −80°C storage and after thawing on ice, treated with lysis buffer in order to isolate the plasma fraction. This step was required because repeated freeze/thawing of the blood at −80°C leads to some cell lysis, releasing haemoglobin into the plasma fraction and inhibiting proper separation by pipetting and visible plasma fraction. To ensure the samples contained no intact erythrocytes, they were incubated in ACK lysing buffer (Invitrogen) at a ratio of 10:1 sample to buffer volume for 3 min at room temperature. This step was followed by centrifugation of the samples at 4°C and 2,000 rpm for 5 min. Following this, the supernatant was removed and stored in 55μL aliquots back at −80°C until required for assays.

The prepared samples were transported to collaborators on dry ice and immediately placed at −80°C upon arrival.

### Cytokine multiplex assay

Based on prior studies that established a link either *in vitro* or *in vivo* with malaria infection, 20 cytokines were selected to evaluate in this study [11-19]. These included; GM-CSF, IFN-γ, IL-1b, IL-2, IL-4, IL-6, IL-8, IL-10, TNF, IL-12(p70), MCP-1, IL-5, TGF-α, TGF-β, IL-12(p40), IL-13, IL-17-α, EGF, M-CSF, and IFN-β. Cytokine quantification was conducted using the Procarta® Cytokine Assay Kit (Affymetrix, Santa Clara, CA, USA) [20]. The final concentrations of cytokine and chemokines are reported in pg/mL.

### Angiopoietin-1/2 ELISA assay

Based on prior studies establishing a link between severe malaria and angiopoietins, Ang-1 and Ang-2 were also evaluated [12,19].

To do this, 96-well plates were coated with 50 μl of capture antibody (R&D; Ang-1: cat. DY923, Ang-2: cat. DY623) per well and left to incubate overnight at room temperature. Plates were then washed seven times with 0.05% Tween-20 in PBS pH 7.2–7.4, and blotted on paper towel. Two hundred μl of reagent diluent (PBS pH 7.2–7.4/0.1% BSA mixture) was then applied to each well and left to incubate for 2 h. Afterwards, the diluent was drained and 100 μl of standard solution (R&D; part 940685) diluted in reagent diluent was applied to standard wells at the top of the standard lanes. The remaining wells in the standards lane were filled with standards serially diluted, so that each well contained half the concentration of standards compared to the well above it, with the last lane containing only reagent diluent. The remaining wells were filled with 50 μl of sample diluted at a ratio of 1:5. The plates were then left to incubate at 4°C overnight.

Ninety-six-well plates were then washed seven times with 0.05% Tween-20 in PBS pH 7.2–7.4, and blotted on paper towel. Fifty μl of detection antibody (R&D: part 840684) reconstituted in reagent diluent was then applied to each well and left to incubate for 2 h at room temperature. Plates were then washed seven times with 0.05% Tween-20 in PBS pH 7.2–7.4, and then blotted on paper towel. Fifty μl of extravidin alkaline phosphatase (Sigma; cat. E2636) that was diluted 1:1,000 in reagent diluent was applied to each well and left to incubate for 45 min at room temperature. Plates were washed another seven times with 0.05% Tween-20 in PBS pH 7.2–7.4, then rinsed with distilled water and blotted on paper towel. One hundred μl of PNPP Sigma fast (Sigma; cat. N2770) was then applied to each well and the plate was left to develop in the dark until ready. The top standards well should be between two and three at an absorbance of 405 nm. The optical density was then determined at a wavelength between 405–570 nm, and a final cytokine concentration was determined in pg/mL.

### Statistical analysis

Cytokine and Ang-1/2 data were broken down into various categories for analysis. The primary focus was on significant differences between FB and CB data using an unpaired student’s *t*-test. Additional comparisons were made for the data set based on patient travel history, age, gender, and species of infection. Samples for which cytokine or Ang-1/2 testing did not yield data due to technical difficulties were excluded. Each comparison was completed using the student’s unpaired *t*-test to assess significant differences between the related data sets defined by a *p* value <0.05 ( Additional file 1: Table S1). The values are reported as mean ± SD. Data analysis was completed using GraphPad Prism (La Jolla, CA, USA) graphing software.

## Results

### Immune response by birth status

**Figure 1:**
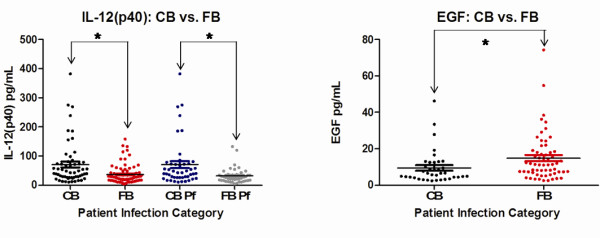
**Canadian-born (CB) and Foreign-born (FB) significant differences**. Significant differences for IL-12 (p40) and EGF were observed between CB and FB travellers. Each coloured circle represents a single patient, with a horizontal line through each data set indicating the mean concentration. Data sets are compared to one another by arrows spanning between them along with an asterix. See Table 2 for *p* values.

The cytokine levels indicated a variety of significant differences when the data was analysed by CB *vs* FB, gender, travel history, and ages grouped by 0–18, 19–55, and 55+ (Table 2). When divided into CB and FB groups, a significantly higher level of IL-12 (p40) was found in CB travellers than in FB travellers (*p* < 0.01) (Figure 1). When the *P. falciparum*-infected patients were examined alone, the CB travellers also had a significantly higher level of IL-12 (p40) compared to FB travellers (*p* = <0.01). A significantly higher level of EGF (*p* = 0.0315) was found to exist in FB compared to CB travellers (Figure 1).

**Table 2:** Statistical summary for comparisons made in this study

**Analysis**	**Marker**	**Data Set 1**	**Mean Set 1**	**Data Set 2**	**Mean Set 2**	***P*** **Value**
**CB** ***vs*** **FB**	Il-12(p40)	CB n = 57	70.39 ± 9.95	FB n = 87	36.24 ± 3.28	<0.01
	EGF	CB n = 37	9.477 ± 1.52	FB n = 60	14.82 ± 1.68	0.0315
**Pf CB** ***vs*** **Pf FB**	IL-12(p40)	CB n = 46	70.31 ± 11.79	FB n = 42	31.53 ± 4.09	<0.01
**Pv Age: 55+** ***vs*** **19-54**	IL-6	55+ n = 10	858.8 ± 454.3	19-54 n = 21	134.7 ± 82.59	0.0366
	MCSF	55+ n = 10	45.66 ± 8.25	19-54 n = 23	31.21 ± 2.82	0.0433
**Species: Pf** ***vs*** **Pv**	MCP-1	Pf n = 86	63.78 ± 11.44	Pv n = 38	392.5 ± 95.45	<0.01
	M-CSF	Pf n = 69	17.61 ± 2.05	Pv n = 38	35.6 ± 3.38	<0.01
	Angiopoietin 2	Pf n = 96	1120 ± 196.5	Pv n = 59	336.5 ± 88.21	<0.01
**Species: Pv** ***vs*** **Po**	IL-12 (p40)	Pv n = 38	51.4 ± 6.76	Po n = 12	26.4 ± 3.73	0.0478
**Species: Pf CB** ***vs*** **Pv CB**	MCP-1	Pf CB n = 46	74.74 ± 16.63	Pv CB n = 8	563.4 ± 233.2	<0.01
	MCSF	Pf CB n = 39	19.59 ± 2.89	Pv CB n = 8	34.37 ± 5.59	0.0367
**Species: Pf FB** ***vs*** **Pv FB**	MCP-1	Pf FB n = 40	51.18 ± 15.43	Pv FB n = 30	347 ± 104.3	<0.01
	M-CSF	Pf FB n = 30	15.32 ± 1.87	Pv FB n = 30	35.3 ± 4.05	<0.01
	IL-12 (p40)	Pf FB n = 42	31.53 ± 4.09	Pv FB n = 30	47 ± 7.01	0.0467
	Angiopoietin 2	Pf FB n = 48	1033 ± 296.6	Pv FB n = 49	310.8 ± 97.81	<0.01
**Travel: West Africa** ***vs*** **other African regions**	IFN-γ	West n = 27	11.88 ± 1.98	Other n = 12	22.5 ± 6.16	0.0414
	IL-6	West n = 22	31.58 ± 6.28	Other n = 12	120.1 ± 56.27	0.0426

Only data for significant differences are shown. Sample size, mean values with standard deviation, and the *p*-value are displayed for all data comparisons that showed significant differences. All other cytokine comparisons were not significantly different.

### Immune response by age and species

**Figure 2:**
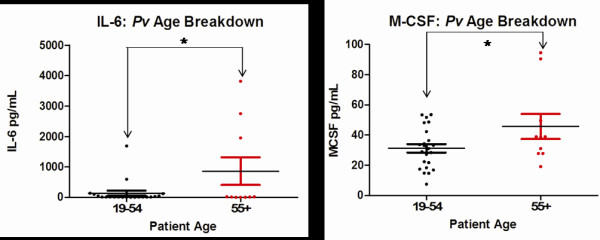
**Age-based significant differences.** Significant differences for IL-6 and M-CSF were observed between 19–54 year olds and those 55 and older who were infected with *Plasmodium vivax.* Each coloured circle represents a single patient, with a horizontal line through each data set indicating the mean concentration. Data sets are compared to one another by arrows spanning between them along with an asterix. See Table 2 for *p* values.

When grouped by ages, *P. vivax* infections indicated a tendency for older adults to have a more robust immune response than younger adults for two different cytokines. Those individuals aged 55 and over demonstrated a higher level of IL-6 (*p* = 0.0366), and M-CSF (*p* = 0.0433), than those aged 19–55 (Figure 2).

**Figure 3:**
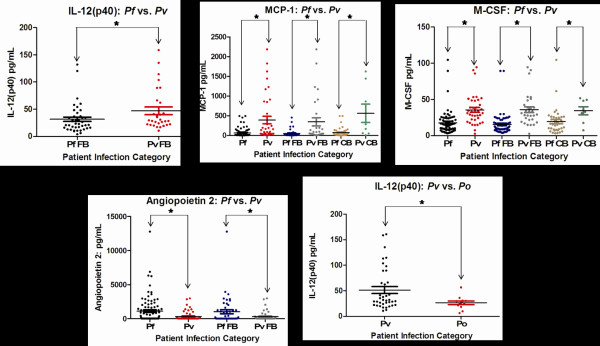
**Species-based significant differences.** Significant differences for IL-12 (p40), MCP-1, M-CSF, and Ang-2 were observed between patients infected with P*lasmodium vivax* and P*lasmodium falciparum*, and a significant difference was noted for IL-12 (p40) between patients infected with *P. vivax* and P*lasmodium ovale.* Each coloured circle represents a single patient, with a horizontal line through each data set indicating the mean concentration. Data sets are compared to one another by arrows spanning between them along with an asterix. See Table 2 for *p* values.

Cytokine responses for individuals infected with *P. ovale, P. malariae, P. vivax, and P. falciparum* were compared and significant differences in the mean level of several cytokines were found when looking at all patient data, comparisons of CB patients only, and comparisons FB patients only. Patients infected with *P. vivax* had higher levels of MCP-1 (*p* = <0.0001) and M-CSF (*p* = <0.0001) than patients infected with *P. falciparum.* Patients infected with *P. vivax* had higher levels of IL-12 (p40) (*p* = 0.0478) than patients infected with *P. ovale*. CB patients infected with *P. vivax* also had higher levels of MCP-1 (*p* = <0.0001), and M-CSF (*p* = 0.0367), when compared to CB patients infected with *P. falciparum.* FB patients infected with *P. vivax* had higher levels for MCP-1 (*p* = 0.0020), M-CSF (*p* = <0.0001), and also IL-12(p40) (*p* = 0.0467), compared to FB patients infected with *P. falciparum* (Figure 3).

### Immune response by region of travel

**Figure 4:**
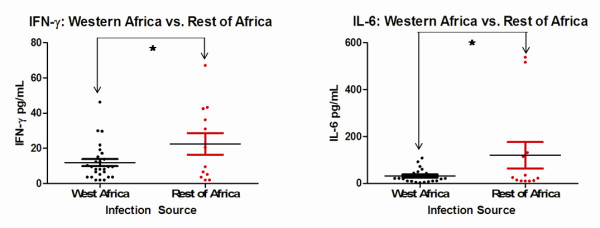
**Significant differences based on travel history.** Significant differences for IFN-*γ* and IL-6 were observed between patients who travelled to west Africa and those who travelled in a different region of Africa. Each coloured circle represents a single patient, with a horizontal line through each data set indicating the mean concentration. Data sets are compared to one another by arrows spanning between them along with an asterix. See Table 2 for *p* values.

Levels of IFN-γ (*p* = 0.0414) and IL-6 (*p* = 0.0426) were significantly lower in patients who travelled to West Africa compared to those who had travelled to other regions in Africa (Figure 4).

### Endothelium activation based on angiopoietin levels

The Ang-1/2 concentrations showed no significant difference between FB and CB patient groups. Additional analysis by gender, travel history, and ages grouped by 0–18, 19–55, and 55+, showed no significant differences for either Ang-1 or Ang-2 levels in the patient plasma samples. A significantly higher level of Ang-2 (*p* < 0.01) was observed in patients infected with *P. falciparum*, compared to those infected with *P. vivax.* The Ang-2 levels were also significantly different (*p* = 0.0218) between the FB patients infected with each species, with a higher mean present for FB *P. falciparum* than for FB *P. vivax* (Figure 3).

## Discussion

This study was conducted on returning travellers who contracted malaria in order to evaluate the cytokine, chemokine, and endothelial activation response in human infection. We hypothesized that in cities like Toronto travellers infected with malaria who are CB will elaborate a different immune response to those who are FB. This response was surmized to be a function of immune memory and protective factors for those who had been previously exposed. The data highlights that IL-12 (p40) and EGF are potential markers of disease severity. It was noted that IL-12 (p40) was significantly increased and EGF significantly decreased in CB. If CB are more vulnerable to severe diseases as has been suspect clinically, then high IL-12 (p40) and low EGF may predict worse outcomes. Consistent with this observation, IL-12 (p40) is a pro-inflammatory cytokine produced by mononuclear phagocytes that activates T-helper 1 cells (Th1) and is important for assisting in innate immune responses against intracellular microbes [14]. However, some studies have shown elevated plasma levels of IL-12 (p40) attributed to mild cases of malaria, with low levels being associated with severe malaria [14,21]. Other studies counter these claims and indicate elevated levels have been seen in individuals with severe malaria in certain Asian populations [13,22]. Additionally, a pre-disposition to severe malaria has been shown to be caused by certain promoter variants that cause an increase in IL-12 production [22,23]. The findings provide support to the idea that IL-12 is more abundant in severe malaria. Further studies into the genetic properties of the IL-12 (p40) promoter in Canadian populations may aid in determining the connections between this study and the findings of others.

EGF binds to the epidermal growth factor receptor (EGFR) where it plays a key role in regulating cellular differentiation, proliferation, and survival [24]. EGF-like domains on *P. falciparum* surface proteins have been studied in great detail for vaccine research [16,24,25], but studies looking at the relationship between EGF levels circulating in the body and malaria infection have not been explored in any detail. The findings indicate a tendency for FB individuals to have a significantly higher level of circulating plasma EGF than CB individuals, but further research will need to be completed to determine the significance of this result and the potential relevance increased levels may have in protecting against severe disease.

Studies focusing on older adults [26], as well as African-based epidemiological data for children [27] show a tendency for these groups to be stricken with malaria more frequently than other age groups, with mortality often at the highest among young children. The results indicated that in *P. vivax* infections, adults over age 55 had more pronounced IL-6 and M-CSF production. IL-6 is a pro-inflammatory cytokine. It is produced by numerous cell types including monocytes, endothelial cells and fibroblasts [28] and has a variety of biological roles, including the stimulation of B and T cell differentiation [29]. IL-6 has been shown to be elevated in Mali children with severe *P. falciparum* malaria [30], in a study of severe cases of malaria among 40 adults aged 21–65 [28], and has been found elevated in patients with cerebral malaria and renal failure [31]. The findings corroborate these studies, suggesting further potential of IL-6 as a marker of disease severity. It was surprising to not find the same result in *P. falciparum* infections, where the role of IL-6 was expected to be more pronounced.

Macrophage colony-stimulating factor (M-CSF) enhances macrophage activity, and it has been suggested that when elevated in malaria, it may lead to an increase in macrophage-mediated platelet destruction [17]. Elevated levels of M-CSF have been attributed to severe cases of *P. falciparum* malaria, where platelet destruction and the release of P-selectin plays a role in thrombocytopaenia associated with severe disease [17]. M-CSF has also been noted to be elevated in *P. vivax* cases [17], so the results indicating a higher level of M-CSF in older patients with *P. vivax* infection appear reasonable. It was noted that when all patient data was divided by species of infection, *P. vivax* had a higher mean cytokine response for M-CSF than *P. falciparum.* This corresponds for the tendency of *P. vivax* to elicit a stronger immune response in patients [32], though this is not necessarily a marker of severity, but is a marker of interest. The significant difference was also seen between M-CSF of just FB patients with *P. vivax* compared to *P. falciparum*, as well as just CB patients.

Monocyte chemo-attractant protein 1 (MCP-1) is required for migration of monocytes and macrophages to sites of inflammation [33], and has been associated with several neuro-inflammatory, and other inflammatory diseases, including HIV-1 encephalitis, and rheumatoid arthritis [34,35]. MCP-1 studies previously showed it to have no association with severe and cerebral malaria in *P. falciparum-*infected patients from Thailand and India [15,36]. The results indicated a substantially higher level of MCP-1 in patients infected with *P. vivax* compared to *P. falciparum* within the full data set, and in comparisons of just FB individuals, and just CB individuals. This finding again corresponds to the tendency of *P. vivax* to have a greater inflammatory response than *P. falciparum*, as does the results with just FB blood samples where the *P. vivax* patients again had a higher level of IL-12 (p40) than *P. falciparum* patients. The increased levels of circulating IL-12 (p40) in *P. vivax* compared to *P. ovale* again attests to the substantial inflammatory response associated with *P. vivax.*

Infections obtained through travel to different areas of Africa also showed a significant difference in patient immune response. A substantial amount of the *P. falciparum* blood samples were from travellers who had been to Western Africa, with most having visited Ghana and Nigeria. All those individuals who had contracted malaria in Western African countries (which included Nigeria and Ghana), were grouped and compared against travellers who were known to have been in other regions of Africa. Significantly lower levels of IL-6 and IFN-γ were found in the Western Africa subset. One study has indicated that there can be reductions in total cases of severe malaria with increasing transmission rates [37], possibly indicating increased transmission of malaria in Western Africa triggers a lower intensity immune response. An additional possibility could be the presence of unique strains in West Africa triggering a different immune response compared to other African strains.

Of the cytokines that were found to be associated with travel, Il-6 has been shown to often be elevated in severe cases of malaria [28,30,31] as mentioned previously. It was also observed to be a contributor to disease outcome in non-endemic areas of India compared to endemic areas [38]. Studies on children in Sudan, an area of unstable malaria transmission, indicate a tendency for severe cases of *P. falciparum* malaria to have elevated levels of IFN- γ [39]; this is also observed in severe cases of *P. falciparum* malaria in India among patients aged five to 75 [13]. Studies on *P. vivax-*infected patients from the Brazilian Amazon indicate plasma levels of IFN- γ increase linearly with a gradual augmentation of disease severity [40].

### Activation of endothelium as assessed by Ang-1 and −2 serum levels in malaria infections

Ang-2 levels have been reported to be elevated in cases of complicated severe malaria compared to uncomplicated malaria [12,19]. Ang-2 primes vascular endothelial cells to exogenous cytokines, and at high concentrations can trigger vascular permeability [41,42]. The results indicate that in *P. falciparum* infections, a higher level of Ang-2 is present than in *P. vivax* infections. These findings appeared in a comparison of all the patient data, as well as in a comparison of just FB patients with *P. falciparum* compared to FB with *P. vivax. P. falciparum* is generally considered to be more virulent than other causes of malaria, and has been observed to more commonly lead to severe cases of malaria [43]. Higher levels of Ang-2 in *P. falciparum* infections are consistent with this endothelium activation factor as Ang-2 and *P. falciparum* are both associated with more severe disease instances. A previous study reported that Ang-2 was more strongly elevated in the peripheral blood of individuals with *P. vivax* malaria compared to individuals with *P. falciparum* infection [44], which corresponds to its tendency to trigger a stronger cytokine response in infected patients.

## Conclusion

The significantly higher mean levels of IL-12 (p40) and significantly lower levels of EGF found in CB travellers may serve to be useful prognostic markers of disease severity and help guide clinical management upon their return. IL-6 and M-CSF in older adults, MCP-1, IL-12(p40) and M-CSF in *P. vivax-*infected patients, and Ang-2 in *P. falciparum-*infected patients may also prove useful in understanding age-associated and species-specific host immune responses. Regional differences in host immune response to malaria infection within the same species (*P. falciparum)* may be related to unique strains circulating in West Africa.

## Abbreviations

CB = Canadian-born; FB = Foreign-born; Pf = Plasmodium falciparum; Pv = Plasmodium vivax; Po = Plasmodium ovale; Pm = Plasmodium malariae.

## Competing interests

The authors declare that they have no competing interests.

## Authors’ contributions

GM and RM performed the experiments, carried out the data analysis, and GM wrote the initial draft of the manuscript. RL assisted with sample preparation and helped to coordinate the study. JK preformed the cytokine multiplex assays. NR carried out the Angiopoietin ELISA assays. DRP, HZ, and WCL conceived the study, and participated in its design and coordination. All authors read and approved the final manuscript.

## Supplementary Material

Additional file 1 All comparisons made between various subsets of the data look for significant differences. Those italicized in red were significant, all others were not significant. Blanks represent instances where insufficient data was present (~1–5 data points available) to make relevant comparisons. Click here for file
