# Supplementary material for: Host immune response in returning travellers infected with malaria
Source: Malar J. 2012 May 3;11:148. doi: 10.1186/1475-2875-11-148 (PMC3586951; doi:10.1186/1475-2875-11-148)
Supplement: Additional file 1 — All comparisons made between various subsets of the data look for significant differences. Those italicized in red were significant, all others were not significant. Blanks represent instances where insufficient data was present (~1–5 data points available) to make relevant comparisons. [file 1475-2875-11-148-S1.docx]

| Supplementary Table 1 | | | | | | | | | | | | | | | | | | | | | | |
| --- | --- | --- | --- | --- | --- | --- | --- | --- | --- | --- | --- | --- | --- | --- | --- | --- | --- | --- | --- | --- | --- | --- |
| **Comparison** | **Ang 1** | **Ang 2** | **G-MCSF** | **IFN-γ** | **Il-1-**β | **IL-2** | **IL-4** | **IL-6** | **IL-8** | **IL-10** | **TNF-α** | **IL-12(p70)** | **MCP-1** | **IL-5** | **TGF-α** | **TGF-**β | **IL-12(p40)** | **IL-13** | **IL-17-α** | **EGF** | **M-CSF** | **IFN-β** |
|  |  |  |  |  |  |  |  |  |  |  |  |  |  |  |  |  |  |  |  |  |  |  |
| **CB vs. FB** | 0.203 | 0.0856 |  |  | 0.2421 |  | 0.304 | 0.2474 | 0.6559 | 0.8189 | 0.2177 | 0.5758 | 0.8132 | 0.0613 | 0.3455 | 0.3949 | ***0.0002*** |  |  | ***0.0315*** | 0.2501 | 0.5838 |
| **CB Pf vs. FB Pf** | 0.0757 | 0.6612 |  | 0.1607 | 0.4585 | 0.5427 | 0.446 | 0.2257 | 0.5648 | 0.4576 | 0.3278 | 0.4201 | 0.3068 | 0.3696 | 0.5712 | 0.3988 | ***0.0036*** |  |  | 0.4563 | 0.3553 | 0.6267 |
| **CB Pv vs. FB Pv** | 0.412 | 0.5236 |  |  | 0.8873 |  | 0.4848 | 0.9801 | 0.6722 | 0.6902 |  | 0.5758 | 0.3624 | 0.1462 | 0.171 | 0.4651 | 0.2134 |  |  | 0.9045 | 0.8534 |  |
| **Male vs. Female** | 0.3519 | 0.7418 | 0.4147 | 0.5805 | 0.2831 | 0.3803 | 0.4725 | 0.5547 | 0.5014 | 0.8377 | 0.303 | 0.3434 | 0.5217 | 0.9664 | 0.2322 | 0.4234 | 0.4008 | 0.2555 | 0.383 | 0.3719 | 0.4081 | 0.1716 |
| **Male Pf vs. Female Pf** | 0.2495 | 0.3293 |  | 0.6121 |  |  |  | 0.7826 | 0.3927 | 0.7786 | 0.2137 |  | 0.87729 |  | 0.1533 | 0.1831 | 0.5526 |  |  | 0.2753 | 0.1834 | 0.1758 |
| **Pf CB Female vs. PF FB Female** | 0.3804 | 0.6823 |  | 0.8276 |  |  |  | 0.6323 | 0.3513 | 0.2841 | 0.0671 |  | 0.5273 |  |  | 0.961 | 0.2351 |  |  | 0.676 | 0.1842 | 0.4122 |
| **Pf CB Male vs. Pf FB Male** | 0.2117 | 0.9966 |  | 0.0844 |  |  |  | 0.326 | 0.2521 | 0.8273 | 0.4625 |  | 0.4307 |  |  | 0.7811 | 0.5854 |  |  | 0.6676 | 0.5123 | 0.3943 |
| **Pf CB Female vs. Pf FB Male** | 0.4261 | 0.6085 |  | 0.3639 |  |  |  | 0.9182 | 0.653 | 0.504 | 0.1207 |  |  |  |  |  | 0.9407 |  |  |  |  |  |
| **Pf FB Female vs. Pf FB Male** | 0.8268 | 0.4684 |  | 0.4313 |  |  |  | 0.3869 | 0.1013 | 0.4607 | 0.8836 |  |  |  |  |  | 0.4621 |  |  |  |  |  |
| **Male Pv vs. Female Pv** | 0.8196 | 0.4691 |  |  | 0.3918 |  |  | 0.2254 | 0.4747 | 0.9873 |  |  | 0.835 |  |  |  | 0.2289 |  |  | 0.0814 | 0.8004 |  |
| **Pv CB Female vs. Pv FB Female** | 0.7966 |  |  |  |  |  |  |  |  |  |  |  |  |  |  |  |  |  |  |  |  |  |
| **Pv CB Male vs. Pv CB Male** | 0.1179 | 0.4463 |  |  |  |  |  |  |  |  |  |  |  |  |  |  |  |  |  |  |  |  |
| **Pv FB Female vs. Pv FB Male** | 0.5367 | 0.4149 |  |  |  |  |  |  |  |  |  |  |  |  |  |  |  |  |  |  |  |  |
| **Age 0-18 vs 19-54** | 0.1904 | 0.2908 |  |  |  |  |  | 0.8589 | 0.5438 | 0.4225 |  |  | 0.924 |  |  |  | 0.9472 |  |  |  | 0.6591 |  |
| **Age 0-18 vs 55+** | 0.438 | 0.967 |  |  |  |  |  | 0.2161 | 0.3254 | 0.5528 |  |  | 0.3836 |  |  |  | 0.3508 |  |  |  | 0.9567 |  |
| **Age 19-54 vs 55+** | 0.5439 | 0.2346 |  |  |  |  |  | 0.1996 | 0.9533 | 0.1024 |  |  | 0.1177 |  |  |  | 0.1792 |  |  |  | 0.6551 |  |
| **Age 0-18 vs. 19+** | 0.2289 | 0.2622 |  |  |  |  |  | 0.6389 | 0.5108 | 0.7955 |  |  | 0.8065 |  |  |  | 0.8086 |  |  |  | 0.7507 |  |
| **Age Under 55 vs 55+** | 0.716 | 0.7723 |  |  |  |  |  | 0.1505 | 0.9335 | 0.1116 |  |  | 0.1199 |  |  |  | 0.152 |  |  |  | 0.751 |  |
| **Pf Age 0-18 vs. 19+** | 0.5614 | 0.3991 |  | 0.5532 |  |  |  | 0.8655 | 0.4329 | 0.368 |  | 0.6065 | 0.6815 |  | 0.5056 |  | 0.8383 |  |  |  | 0.6941 | 0.5979 |
| **Pf Age 0-18 vs. 55+** | 0.3671 | 0.1225 |  | 0.8443 |  |  |  | 0.7179 | 0.6208 | 0.8342 |  | 0.2303 | 0.5383 |  | 0.1432 |  | 0.3639 |  |  |  | 0.2324 | 0.3498 |
| **Pf Age 19-54 vs. 55+** | 0.5546 | 0.4647 |  | 0.5309 |  |  |  | 0.8918 | 0.5323 | 0.3114 |  | 0.5834 | 0.1705 |  | 0.1358 |  | 0.1528 |  |  |  | 0.6588 | 0.1011 |
| **Pf Age 0-18 vs. 19+** | 0.4988 | 0.3096 |  | 0.6061 |  |  |  | 0.8351 | 0.4679 | 0.6095 |  | 0.6533 | 0.9068 |  | 0.3588 | 0.8883 | 0.9035 |  |  |  | 0.7412 | 0.5582 |
| **Pf Age Under 55 vs. 55+** | 0.4523 | 0.336 |  | 0.5744 |  |  |  | 0.8537 | 0.6056 | 0.3566 |  | 0.6249 | 0.3588 |  | 0.0659 | 0.5959 | 0.1346 |  |  |  | 0.6771 | 0.0576 |
| **Pv Age 0-18 vs. 19+** | 0.2049 |  |  |  | 0.1681 |  |  | 0.8015 | 0.2491 | 0.7268 |  |  | 0.8853 |  |  |  | 0.7862 |  |  | 0.4893 | 0.6177 |  |
| **Pv Age 0-18 vs. 55+** | 0.3259 |  |  |  | 0.4707 |  |  | 0.3147 | 0.6973 | 0.3331 |  |  | 0.7859 |  |  |  | 0.6335 |  |  | 0.425 | 0.534 |  |
| **Pv Age 19-54 vs. 55+** | 0.242 | 0.7398 |  |  | 0.29 |  |  | ***0.0366*** | 0.0748 | 0.0891 |  |  | 0.4823 |  |  |  | 0.8128 |  |  | 0.8809 | ***0.0433*** |  |
| **Pv Age 0-18 vs. 19+** | 0.2592 |  |  |  | 0.4653 |  |  | 0.545 | 0.8081 | 0.5317 |  |  | 0.9813 |  |  |  | 0.7253 |  |  | 0.4544 | 0.9912 |  |
| **Pv Age Under 55 vs. 55+** | 0.3904 | 0.9784 |  |  | 0.1935 |  |  | ***0.021*** | 0.0825 | 0.0564 |  |  | 0.5071 |  |  |  | 0.7484 |  |  | 0.7469 | 0.0745 |  |
| **Pf vs. Pv** | 0.2341 | ***0.0031*** |  |  | 0.5348 |  | 0.5183 | 0.8394 | 0.8608 | 0.5832 |  | 0.713 | ***>0.0001*** | 0.0826 | 0.3571 |  | 0.9705 |  | 0.3883 | 0.617 | 0.7384 |  |
| **Pf CB vs. Pv CB** | 0.7172 | 0.2062 |  |  | 0.7428 |  | 0.7401 | 0.8513 | 0.7134 | 0.8355 |  | 0.7879 | ***>0.0001*** | 0.4154 | 0.6668 |  | 0.9332 |  | 0.6292 | 0.7186 | 0.743 |  |
| **Pf FB vs. Pv FB** | 0.7192 | ***0.0218*** |  |  | 0.2791 |  | 0.5893 | 0.2625 | 0.7534 | 0.3881 |  | 0.291 | 0.002 | 0.2859 | 0.4715 |  | ***0.0467*** |  | 0.4638 | 0.4726 | ***>0.0001*** |  |
| **Pv vs Po** | 0.6173 | 0.9729 |  |  |  |  |  | 0.307 | 0.8069 | 0.2571 |  |  | 0.0739 |  |  |  | ***0.0478*** |  |  | 0.0981 | 0.1503 |  |
| **Pf vs Po** | 0.9081 | 0.1407 |  |  |  |  |  | 0.4717 | 0.96 | 0.2697 |  |  | 0.6774 |  |  |  | 0.1617 |  |  | 0.9814 | 0.7609 |  |
| **Pf West Africa vs. Rest of Africa** | 0.6486 | 0.2087 |  | ***0.0481*** |  |  |  | ***0.0426*** | 0.8024 | 0.8521 | 0.2864 | 0.1731 | 0.2347 |  | 0.4796 | 0.1161 | 0.1013 |  |  | 0.1323 | 0.1835 |  |
| **Pv Central America vs. Middle East** | 0.753 |  |  |  |  |  |  | 0.0934 | 0.3243 | 0.3673 |  |  | 0.3081 |  |  |  | 0.7219 |  |  | 0.7167 | 0.111 |  |
| **Pv Africa vs. Central America** | 0.2552 |  |  |  |  |  |  |  |  |  |  |  |  |  |  |  |  |  |  |  |  |  |
| **Pv Africa vs. Middle East** | 0.5719 |  |  |  |  |  |  |  |  |  |  |  |  |  |  |  |  |  |  |  |  |  |
